# Clinical validity of two different grading systems for lumbar central canal stenosis: Schizas and Lee classification systems

**DOI:** 10.1371/journal.pone.0233633

**Published:** 2020-05-27

**Authors:** Yeon-jee Ko, Eugene Lee, Joon Woo Lee, Chi Young Park, Jungheum Cho, Yusuhn Kang, Joong Mo Ahn

**Affiliations:** Departments of Radiology, Seoul National University Bundang Hospital, Seongnam, Korea; University of Toronto, CANADA

## Abstract

**Objective:**

To assess the learnability of two magnetic resonance imaging (MRI) grading systems for lumbar central canal stenosis based on inter-observer agreement and test-retest reliability of doctors with no prior knowledge of the two systems.

**Materials and methods:**

Two clinical fellows, one novice radiology resident, one neurosurgeon, and one orthopedic surgeon, who were unaware of the two qualitative MRI grading systems prior to this study, acquainted themselves with the teaching files. All five observers independently assessed the LCCS grade of 70 patients using T2-weighted axial magnetic resonance images at the L2-3, L3-4, L3-4, and L5-S1 disc levels. Analysis was performed twice at an interval of two months.

**Results:**

The inter-observer agreement among all five readers was excellent and test-retest reliability was moderate to excellent for both the Schizas and Lee systems. Positive percentage agreements were found to be over 0.8 in almost all observers with relatively narrow 95% confidence limits.

**Conclusion:**

Both Schizas and Lee MRI grading systems for LCCS are reliable grading systems, and can be used as a learnable method for both clinicians and radiologists.

## Introduction

Lumbar central canal stenosis (LCCS) is defined as the narrowing of the central spinal canal caused by degenerative changes with compression of neural and vascular structures, resulting in various degrees of clinical disability [1]. As the population ages and average life expectancy increases, the number of patients with symptomatic LCCS has increased [2]. In order to select the most efficacious therapy, patient’s clinical course, severity of symptoms, and radiological features are usually considered [3].

In fact, MRI plays a key role in the diagnosis of LCCS, and there have been reports of a statistical association between MRI grading and the patient's disability or neurological impairment [4–5]. On the other hand, there have been several reports of poor correlation in patients' clinical symptoms and radiological severity [6–7]. Nevertheless, the assessment of LCCS using grading system is very widely used in clinical practices. However, the problem is that there is no clear consensus on the grading system used in the actual clinical field [8], that the criteria for the diagnosis are inconsistent from those of radiologists and clinicians [9], and that these problems affect the area of research.

Among the various LCCS grading systems proposed so far, the following two magnetic resonance imaging (MRI) grading systems are recently proposed and widely quoted. [10]: Schizas et al. (2010) suggested a 7-grade classification based on the morphology of the dural sac with the rootlet/cerebrospinal fluid (CSF) ratio taken into account [11]; Lee et al. (2011) reported a 4-grade classification system based on the obliteration of CSF space in front of the cauda equina in the dural sac and the separation degree of the cauda equina [1].

Due to the ability to perform rapid visual assessment without requiring specific measurement tools, both qualitative MRI grading systems have been widely used in clinical guidelines and reports as radiological parameters to classify LCCS [12–16]. Additionally, there have been attempts to develop a gold standard classification system (or criteria) for LCCS using the combination of various tools, including MRI grade as a radiological parameter [10]. For the appropriate selection of a standard tool for LCCS classification, comparability and validity of the two MRI grading systems is required to determine interpretability, proficiency, and reproducibility among beginners who are not familiar with the grading systems [10,17]. The purpose of this study was to assess the learnability of the two MRI grading systems for LCCS based on inter-observer agreement and test-retest reliability of doctors with no prior knowledge of the two systems.

## Materials and methods

### Lumbar MRI cases

This study was approved by the institutional review board of Seoul National University Bundang Hospital (No.: B-1608-360-102), and informed consent was waived due to the retrospective nature of this study. This paper has implemented English language editing in Editage (www.editage.co.kr).

Among patients who visited our institution due to back pain and/or radiculopathy, 70 lumbar spine MRI studies performed at our institution or outside hospitals during March 2016 were consecutively selected after excluding patients meeting any of the following criteria: 1) non-degenerative disease such as infection, fracture, or tumor; 2) past spinal surgical history; 3) lack of T2-weighted axial image at any of the L2-3, L3-4, L3-4, or L5-S1 disc levels. Using the lowest ICC value with statistical significance in the levels evaluated in the study (0.730, [1]), the required sample size was calculated as 45 when the precision was set as 0.1 with five raters [18].

Among a total 70 patients, 25 were men and 45 were women. Patient age ranged from 23 to 87 years, with a mean age of 65.8 years. Patient characteristics and imaging features are described in Table 1.

**Table 1:** Patient characteristics (n = 70).

Characteristics		Case (%)
Age (years)	65.8 ± 14.9	
Sex	Male	25 (35.7%)
	Female	45 (64.3%)
Symptoms	Low back pain	33 (47.1%)
	Buttock pain	40 (57.1%)
	Radicular pain	53 (75.7%)
	Weakness	6 (8.6%)
Imaging features	Lumbar scoliosis	36 (51.4%)
	Lumbar kyphosis	6 (8.6%)
	Degenerative spondylolisthesis	
	L2/3	2 (2.9%)
	L3/4	13 (18.6%)
	L4/5	25 (35.7%)
	L5/S1	1 (1.4%)
	Spondylolytic spondylolisthesis	
	L4/5	1 (1.4%)
	L5/S1	4 (5.7%)
	Retrolisthesis	
	L2/3	18 (25.7%)
	L3/4	19 (17.1%)
	L4/5	11 (15.7%)
	L5/S1	14 (20.0%)
	Combined HIVD to the central canal	
	L2/3	4 (5.7%)
	L3/4	9 (12.9%)
	L4/5	30 (42.9%)
	L5/S1	23 (32.9%)

HIVD: Herniated intervertebral disc.

### MRI grading systems for lumbar central canal stenosis

**Fig 1:**
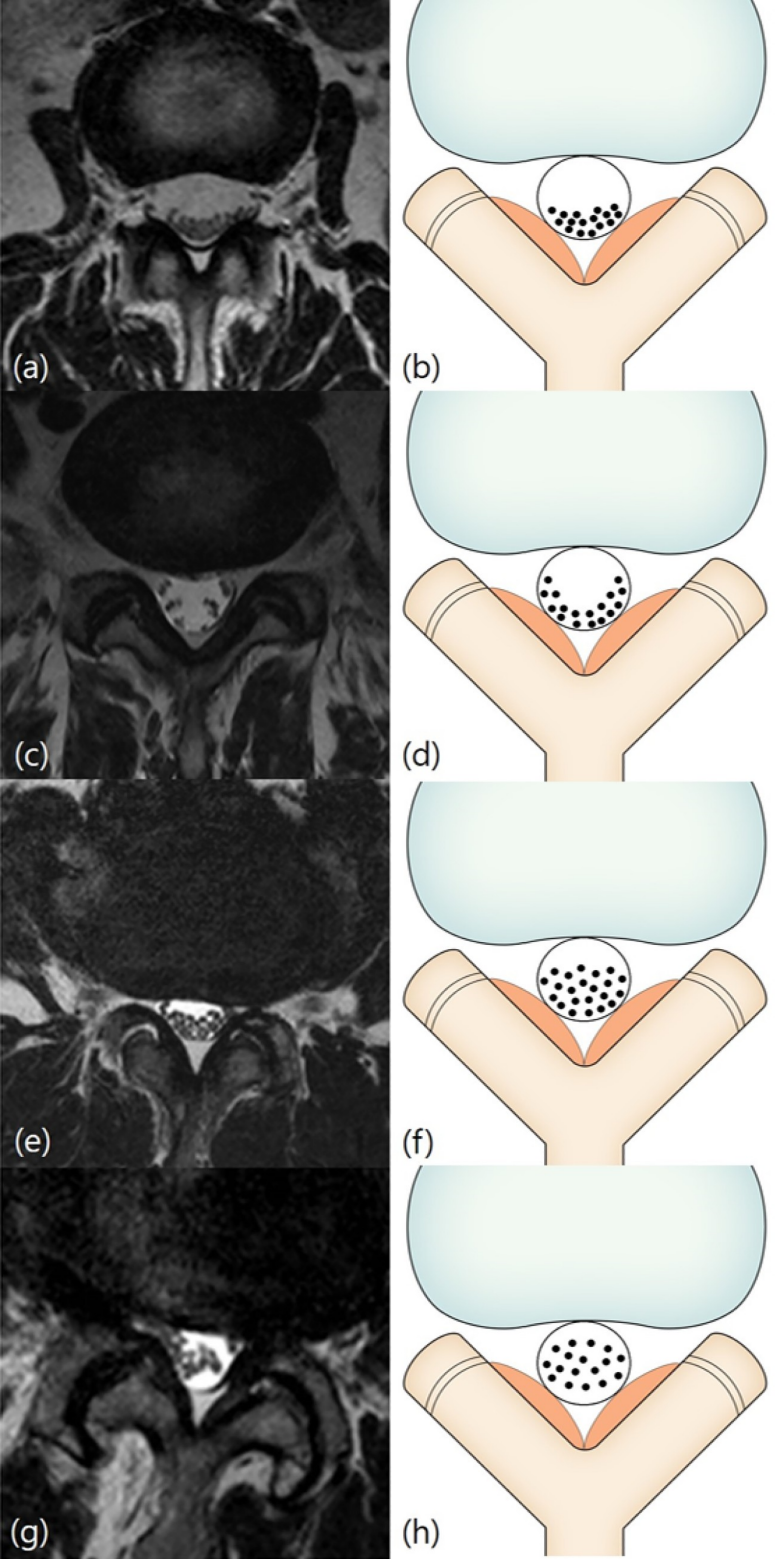
Schizas classification. Grade A (no or minor stenosis). Fig 1A and 1B: Grade A1 (no or minor stenosis). A 59-year-old woman with right lower extremity radiculopathy. T2-weighted axial magnetic resonance image at L2/3 disc level shows the rootlets lying dorsally and occupying less than half of the dural sac area. Fig 1C and 1D: Grade A2 (no or minor stenosis). A 65-year-old man with pain in both buttocks. T2-weighted axial magnetic resonance image at L5/S1 disc level shows the rootlets lying dorsally with a horseshoe configuration. Fig 1E and 1F: Grade A3 (no or minor stenosis). A 71-year-old man with left lower extremity radiculopathy. T2-weighted axial magnetic resonance image at L3/4 disc level shows the rootlets lying dorsally and occupying more than half of the dural sac area. Fig 1G and 1H: Grade A4 (no or minor stenosis). A 77-year-old man with lower back pain. T2-weighted axial magnetic resonance image at L3/4 disc level shows the rootlets lying centrally and occupying the majority of the dural sac area.

**Fig 2:**
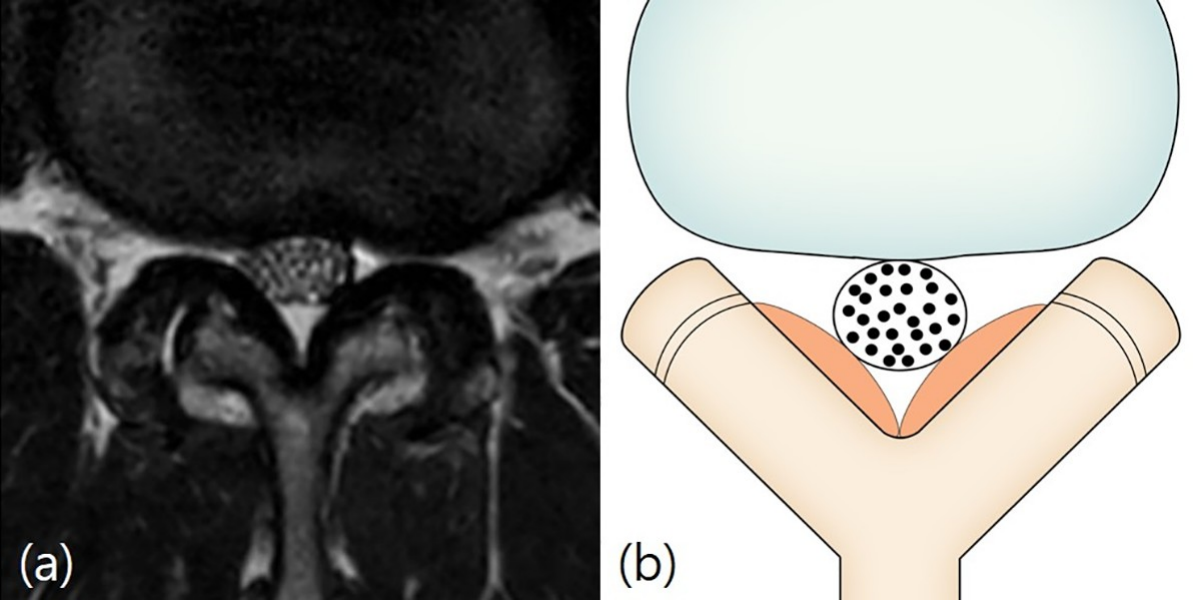
Schizas classification. Grade B (moderate stenosis). Fig 2A and 2B: A 59-year-old man with pain in both buttocks and both lower extremities. T2-weighted axial magnetic resonance image at L2/3 disc level shows the rootlets occupying the entire dural sac, but rootlets can still be individualized.

**Fig 3:**
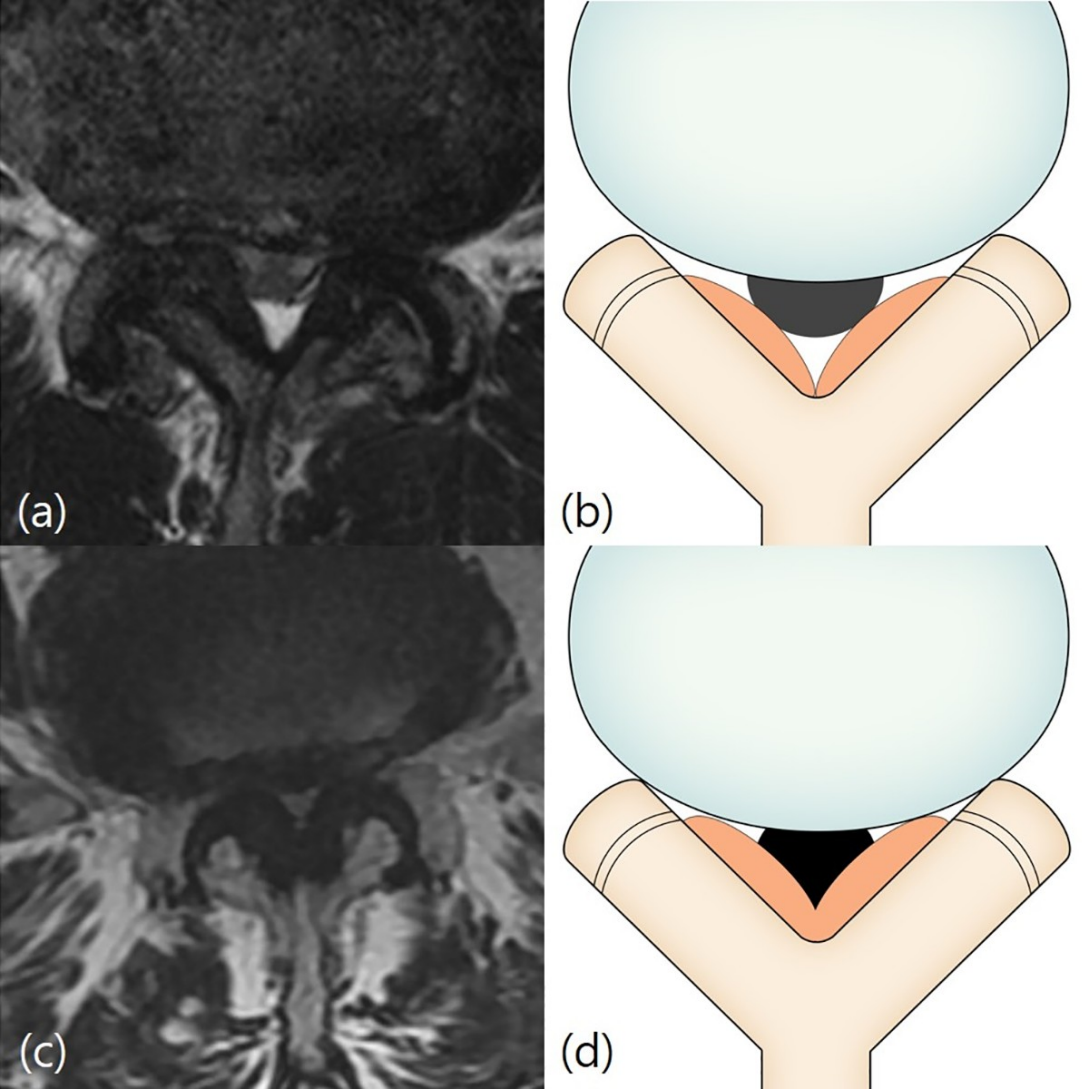
Schizas classification. Grade C and D (severe or extreme stenosis). Fig 3A and 3B: Grade C (severe stenosis). A 71-year-old man with left lower extremity radiculopathy. T2-weighted axial magnetic resonance image at L4/5 disc level shows that no recognizable rootlets with complete effacement of cerebrospinal fluid space, but epidural fat was present posteriorly. Fig 3C and 3D: Grade D (extreme stenosis). A 70-year-old woman with right lower extremity radiculopathy. T2-weighted axial magnetic resonance image at L4/5 disc level shows no recognizable rootlets and no epidural fat posteriorly.

The Schizas system [11] is a 7-grade classification system based on the morphology of the dural sac on T2-weighted axial MRI with the rootlet/CSF fluid ratio taken into account. Grade A, no or minor stenosis, refers to clearly visible CSF inside the dural sac with inhomogeneous distribution. Grade A1 refers to the condition where the rootlets lie dorsally and occupy less than half of the dural sac area (Fig 1A and 1B). Grade A2 refers to cases where the rootlets lie dorsally, in contact with the dura but in a horseshoe configuration (Fig 1C and 1D). Grade A3 refers to rootlets lying dorsally and occupying more than half of the dural sac area (Fig 1E and 1F). Grade A4 refers to cases where the rootlets lie centrally and occupy the majority of the dural sac area (Fig 1G and 1H). Grade B, moderate stenosis, includes cases where the rootlets occupy the entire dural sac, but can still be individualized (Fig 2A and 2B). Grade C, severe stenosis, refers to cases where no rootlets can be recognized, with the dural sac demonstrating a homogeneous gray signal with no visible CSF signal, but epidural fat present posteriorly (Fig 3A and 3B). Grade D, extreme stenosis, refers to no rootlets being recognizable and no epidural fat posteriorly (Fig 3C and 3D).

**Fig 4:**
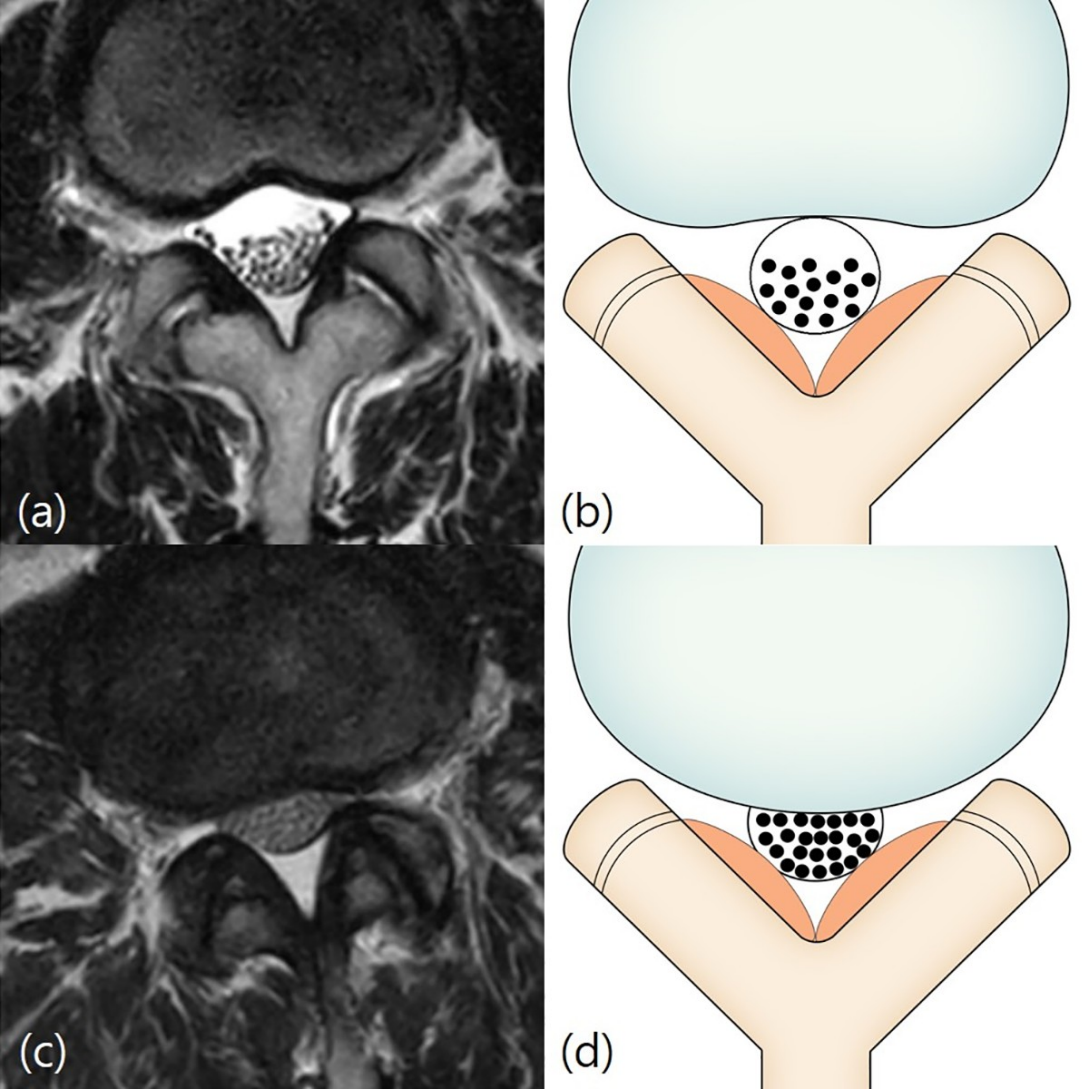
Lee classification. Grade 0 and 1 (no or mild stenosis). Figs 4A and 3B: Grade 0 (no stenosis). A 66-year-old woman with lower back pain. T2-weighted axial magnetic resonance image at L2/3 disc level shows no obliteration of the anterior cerebrospinal fluid space. Fig 4C and 4D: Grade 1 (mild stenosis). A 61-year-old woman with lower back pain. T2-weighted axial magnetic resonance image at L2/3 disc level shows mild obliteration of the anterior cerebrospinal fluid space and all cauda equina clearly separated from each other.

**Fig 5:**
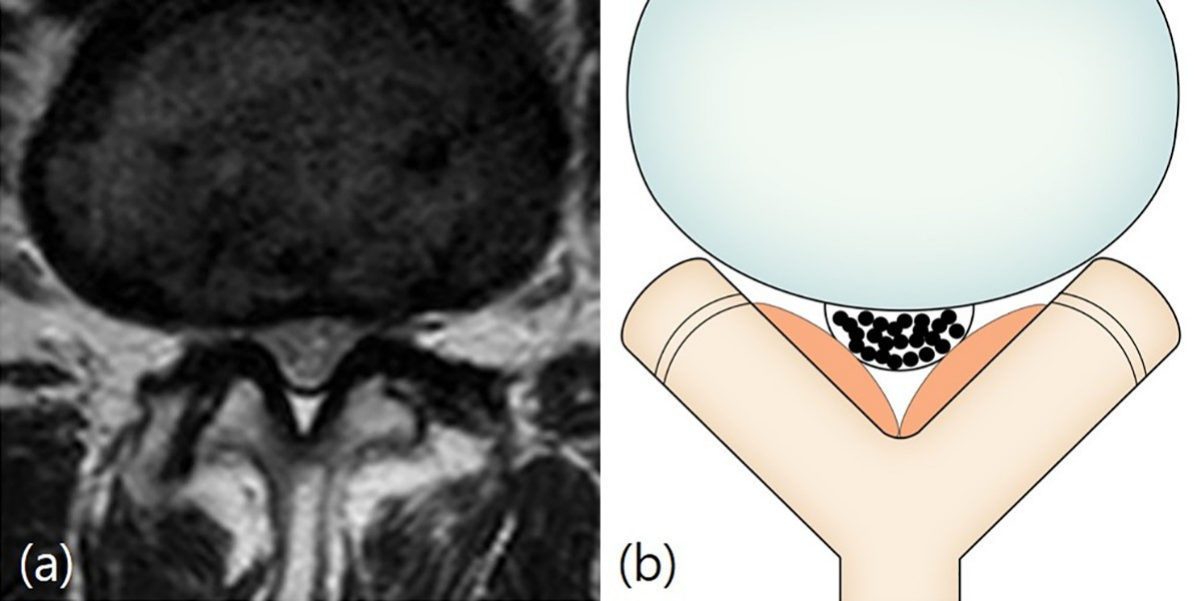
Lee classification. Grade 2 (moderate stenosis). Fig 5A and 5B: A 59-year-old woman with right lower extremity radiculopathy. T2-weighted axial magnetic resonance image at L4/5 disc level shows moderate obliteration of the anterior cerebrospinal fluid space and some cauda equina aggregation.

**Fig 6:**
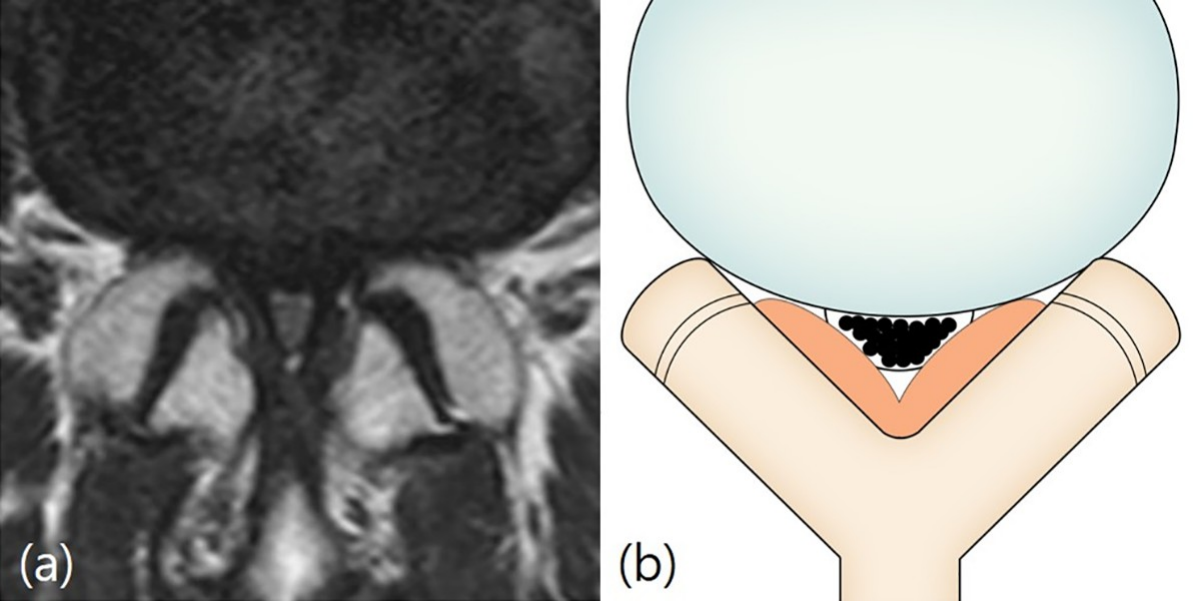
Lee classification. Grade 3 (severe stenosis). Fig 6A and 6B: A 69-year-old woman with pain in both lower extremities. T2-weighted axial magnetic resonance image at L4/5 disc level shows severe obliteration of the anterior cerebrospinal fluid space, marked compression of the dural sac, and the entire cauda equina appearing as one bundle.

The Lee system [1] is a 4-grade classification system based on the degree of separation of the cauda equina on T2-weighted axial MRI. Grade 0, no LCCS, refers to no obliteration of the anterior CSF space (Fig 4A and 4B). Grade 1, mild LCCS, refers to mild obliteration of the anterior CSF space and all cauda equina clearly separated from each other (Fig 4C and 4D). Grade 2, moderate LCCS, refers to moderate obliteration of the anterior CSF space and some cauda equina aggregation where it is impossible to identify each other visually (Fig 5A and 5B). Grade 3, severe LCCS, refers to severe obliteration of the anterior CSF space, marked compression of the dural sac, and the entire cauda equina appearing as one bundle (Fig 6A and 6B).

### Image analysis

Image review was conducted by five observers—two clinical fellows with 3 months of experience, one new radiology resident with one month of experience, one orthopedic surgeon with 14 years of experience, and one neurosurgeon with 10 years of experience. All five observers were unaware of the two grading systems before this study.

In the first phase, two radiologists (one fellow and one professor), who did not participate in the image analysis, created a teaching file that explained the definitions and criteria of two different LCCS MRI grading systems. In the teaching file, the two grading systems were simply designated as ‘type A’ and ‘type B’, blinded to name or authors of the grading systems. All five observers acquainted themselves with the teaching files.

In the second phase, all five observers independently assessed the LCCS grade using T2-weighted axial magnetic resonance images at the L2-3, L3-4, L4-5, and L5-S1 disc levels, and checked the level with the guidance of T2-weighted sagittal image at the median plane. Time interval between the first and the second phase was one month.

In the third phase, after a time interval of two months from the second phase, the same analysis was performed by all observers. Thus, the analysis was performed twice, with the second analysis performed two months after the first analysis, including all cases and both grading systems during each assessment.

### MRI parameters

Lumbar spine MRI exams conducted at our hospital were performed using a 1.5 T magnetic resonance scanner (Gyroscan Intera, Philips Healthcare), or 3.0 T magnetic resonance scanner (Achieva, Philips Healthcare) with a Synergy Spine Coil (Philips Healthcare). Each patient was placed in the supine position with or without utilizing knee bolster. The typical acquisition parameters of lumbar MRI using 1.5T and 3.0T MR systems in our institution are listed in Table 2.

**Table 2:** 1.5T and 3T lumbar MRI acquisition parameters.

T2-weighted FSE sequences				
	1.5T		3T	
	Axial	Sagittal	Axial	Sagittal
**TR (ms)**	2350			2000–3700
**TE (ms)**	88	129	100–120	120
**Matrix size**	320 x 320	512 x 512	256 x 240–256	350–900 x 250–300
**FOV (cm)**	180 x 180	340 x 340	150 x 150	400–610 x 300–350
**Section thickness (mm)**	4	4	4	4
**Echo-train length**	16–17	13–20	24–30	20–30
**No. of acquisitions**	1–2	1–2	1–2	1–2

Sixteen magnetic resonance exams were performed at outside hospitals, where both T2-weighted axial and sagittal images were obtained using 1.5 T magnetic resonance scanners.

### Statistical analysis

Inter-observer agreement among the five readers and test-retest reliability were analyzed using the intra-class correlation coefficient (ICC). ICC reliability was categorized as slight (0–0.20), fair (0.21–0.40), moderate (0.41–0.60), substantial (0.61–0.80), and excellent (0.81–1.00). Positive percentage agreements (PPA) and 95% confidence limits (CL) were obtained between one representative grade and five observers using the Schizas and Lee grading systems to determine the whether the observed counts significantly differ from the expected distribution. This representative grade was composed of one grade showing the highest agreement rate among the grading results of five observers. All statistical analyses were performed with a statistical software program (SPSS23; SPSS, Chicago, Ill, USA). A p-value of less than 0.05 was considered to indicate a significant difference.

## Results

A total of 70 patients were eligible to be included in the analysis, and a total of 280 disc levels with T2-weighted axial magnetic resonance images were analyzed for LCCS grading.

In both the Schizas and Lee grading system, inter-observer agreements showed very similar results (Table 3). The inter-observer agreement of the Schizas grading system among all five readers was excellent at all disc levels in both the second and the third phase analyses. ICC ranged from 0.827 to 0.983. ICCs of each disc level were as follows: 0.946 to 0.965 at L2-3 disc level, 0.974 to 0.983 at L3-4 disc level, 0.954 to 0.959 at L4-5 disc level, and 0.827 to 0.828 at L5-S1 disc level (*p* < 0.001).

**Table 3:** Intra-class correlation coefficient reliabilities for inter-observer agreement of the Schizas and Lee systems.

Classification System	Phase		Lumbar Levels (n = 70)			
			L2-3	L3-4	L4-5	L5-S1
Schizas system [11]	2^nd^ phase	ICC	0.946	0.974	0.959	0.828
	3^rd^ phase		0.965	0.983	0.954	0.827
Lee system [1]	2^nd^ phase	ICC	0.945	0.975	0.962	0.840
	3^rd^ phase		0.956	0.983	0.960	0.853
All p values < 0.001						

* A *p* value less than 0.05 was defined as statistically significant. ICC: intra-class correlation coefficient.

The inter-observer agreement of the Lee grading system between all five readers was also excellent at all disc levels in both the second and the third phase analyses. ICC ranged from 0.840 to 0.983. ICCs of each disc level were as follows: 0.945 to 0.956 at L2-3 disc level, 0.975 to 0.983 at L3-4 disc level, 0.960 to 0.962 at L4-5 disc level, and 0.840 to 0.853 at L5-S1 disc level.

In both the Schizas and Lee grading system, test-retest reliability also showed very similar results (Table 4). Overall test-retest reliability for the Schizas system was moderate to excellent (0.652 to 0.996). ICC values of each disc level were as follows: 0.885 to 0.975 at L2-3 disc level, 0.930 to 0.995 at L3-4 disc level, 0.915 to 0.996 at L4-5 disc level, and 0.652 to 0.839 at L5-S1 disc level.

**Table 4:** Test-retest reliability of the Schizas and Lee systems.

Observers	Classification system		Lumbar Levels (n = 70)			
			L2-3	L3-4	L4-5	L5-S1
Observer 1	Schizas system	ICC	0.885	0.974	0.959	0.828
	Lee system		0.948	0.954	0.943	0.631
Observer 2	Schizas system	ICC	0.838	0.930	0.915	0.839
	Lee system		0.867	0.915	0.874	0.847
Observer 3	Schizas system	ICC	0.948	0.968	0.966	0.798
	Lee system		0.953	0.970	0.924	0.911
Observer 4	Schizas system	ICC	0.975	0.995	0.996	0.771
	Lee system		0.989	0.995	0.963	0.935
Observer 5	Schizas system	ICC	0.953	0.978	0.980	0.721
	Lee system		0.944	0.987	0.983	0.936
All p values < 0.001						

* A *p* value less than 0.05 was defined as statistically significant. ICC: intra-class correlation coefficient.

Overall test-retest reliability of the Lee system was moderate to excellent (0.631 to 0.995). ICC values of each disc level were as follows: 0.867 to 0.989 at L2-3 disc level, 0.915 to 0.995 at L3-4 disc level, 0.874 to 0.983 at L4-5 disc level, and 0.631 to 0.936at L5-S1 disc level.

In the Schizas and Lee grading systems, positive percentage agreements were found to be over 0.8 in almost all observers (Table 5). However in the Schizas grading system, observer 2 showed slightly lower positive percentage agreements at the first reading of L4/5 and the second reading of L5/S1 than those of the other groups, but the positive percentage agreement was 0.771, suggesting a high agreement. In the Lee grading system, observer 2 also showed lower positive percentage agreements in all evaluated disc levels.

**Table 5:** Positive percentage agreements of the Schizas and Lee system.

Observers	Classification system		Lumbar levels (n = 70)			
			L2/3	L3/4	L4/5	L5/S1
Observer 1	Schizas system	PPA (2^nd^ and 3^rd^ phase)	0.900	0.943	0.843	0.971
			0.929	0.929	0.843	0.986
	Lee system		0.857	0.886	0.800	0.914
			0.829	0.929	0.857	0.943
Observer 2	Schizas system	PPA (2^nd^ and 3^rd^ phase)	0.900	0.900	0.771	0.814
			0.971	0.929	0.814	0.771
	Lee system		0.457	0.443	0.657	0.100
			0.400	0.400	0.671	0.071
Observer 3	Schizas system	PPA (2^nd^ and 3^rd^ phase)	0.957	0.871	0.886	0.943
			0.943	0.929	0.857	0.957
	Lee system		0.886	0.857	0.857	0.914
			0.871	0.971	0.871	0.900
Observer 4	Schizas system	PPA (2^nd^ and 3^rd^ phase)	0.929	0.957	0.943	0.943
			0.929	0.943	0.857	0.929
	Lee system		0.929	0.857	0.829	0.914
			0.871	0.900	0.786	0.943
Observer 5	Schizas system	PPA (2^nd^ and 3^rd^ phase)	0.943	0.857	0.829	1.0000
			0.929	0.843	0.829	0.957
	Lee system		0.886	0.886	0.871	0.929
			0.886	0.814	0.829	0.943

PPA: positive percentage agreement.

## Discussion

This study validates the inter-observer agreement and test-retest reliability of five doctors who were unfamiliar with the two qualitative MRI grading systems for LCCS before the study. Inter-observer agreement was excellent for both the Schizas system and the Lee system. Test-retest reliability was moderate to excellent for both the Schizas and Lee system. This study has strengths in two ways: first, it seems to be the only study that has evaluated the reliability of the two classification systems in a comparative fashion and second, the study participants have varying levels of experience and training backgrounds which speak to the generalizability of the study.

Many different radiological quantitative or qualitative criteria have been introduced for evaluation of LCCS. Despite tremendous efforts to establish a broadly accepted classification system, a consensus on normative and clear cutoff values to guide treatment decision-making in LCCS has not yet been reached [10, 17].

Two qualitative MRI grading systems of LCCS were proposed at nearly the same time. The two systems are similar as they evaluate the association between the CSF space and cauda equina [1,11,17]. The Schizas grading system focuses on the CSF/rootlet ratio and effacement of dorsal epidural fat, and showed moderate inter-observer agreement (k = 0.65) [11]. The Lee grading system focuses on obliteration of ventral CSF space and aggregation of the cauda equina, and showed substantial to excellent inter-observer agreement (ICC reliability = 0.730 to 0.953) [1].

Previous reports have evaluated the inter-observer agreements of either of the two MRI grading systems. Weber et al. reported substantial inter-observer agreement using the Schizas system between two highly experienced radiologists and two clinicians (consultant neurosurgeons) [19]. Lønne et al. reported substantial inter-observer agreement between two experienced neuro-radiologists using the Schizas system [20]. Park et al. reported substantial inter-observer agreement between two radiologists (with 12 and 10 years of experience, respectively) using the Lee system, and even higher inter-observer agreement (0.814) in the older age group (≥57 years) than that (0.718) in the younger age group (<57 years) [21]. However, there is no published assessment of LCCS using both MRI grading systems on the same cases. Furthermore, image analysis in previous studies was done by radiologists with significant experience.

Our study applied the two MRI grading systems for LCCS to the same cases. Image analysis was performed by radiologists and clinical fellows with little experience, and outside hospital clinicians who were unaware of the systems prior to the study. The results of this analysis showed higher inter-observer agreement than in previous studies. It could be concluded that these two MRI grading systems are easy to understand for radiologists or clinicians who are not familiar with these. Rapid visual assessment without using specific measurement tools, and accounting for background knowledge on morphology and anatomical variance, may contribute to the strength of these grading systems [10, 20].

Park et al. reported that the highest agreement was found at the L4-5 disc level (k = 0.789), with the highest incidence of stenosis by assessment of LCCS grade using the Lee MRI grading system [21]. In our study, the highest inter-observer agreement was found at the L3-4 disc level and the lowest inter-observer agreement was found at the L5-S1 disc level for both MRI grading systems. This difference may be a result of the low incidence of stenosis at the L5-S1 level in our patients. Among the 70 cases of L5-S1 disc level, none were considered grade D in the Schizas system. There was only one case that received a grade of severe as per the Lee system and grade C in the Schizas system, from only one observer.

Weber et al. reported excellent intra-observer agreement using the Schizas system [19]. In this study, the overall test-retest reliability for both the Schizas and Lee system was moderate to excellent.

In most of the clinical studies the inter-observer differences show less agreement, while the intra-observer agreement is higher just like in the above mentioned studies [11, 19]. However, in our study, test-retest reliability was slightly lower than inter-observer agreement in both grading systems, especially L5/S1 level. Perhaps the study design itself is a qualitative assessment and the experience of observers is so low that the grading of stenosis at the time of repeated evaluation cannot be consistently graded if it is over the borderline. Furthermore, these may result in a somewhat low test-retest reliability in an inexperienced observer with possible interval learning effect, but they do not make a big statistical difference of total grading sets of all observers between 2^nd^ and 3^rd^ phases and as expected, Schizas and Lee grading system maintain similar levels of grading when statistically looking at the entire five observers, which disproves the high inter-observer reliability. If an experienced radiologist conducted grading, it is estimated that the test retest reliability would have been higher than the inter-observer agreement.

In this study, evaluation of positive percentage agreements was carried out because it does not mean good learnability only if there is high inter-observer agreement or test-retest reliability. In the Schizas and Lee grading systems, positive percentage agreements were found to be over 0.8 in almost all observers, suggesting a high agreement rate between the two groups. However, in both grading systems, only observer 2 (spine neurosurgeon) shows lower agreements than other observers. In most cases, grading of stenosis is often evaluated with a high grade compared to other observers which is a reflection of the experience of spinal stenosis grade in his practice. It is also possible that the definition of stenosis grading is difficult to apply to lower lumbar levels especially for L5/S1 level because of the relatively small central canal size and small number in rootlets. This problem can be applied to both grading systems. It is necessary to reflect these problems in a modified grading system in the future.

There are a few limitations in this study. We did not correlate the severity of the two MRI grading systems with clinical symptoms, neurological signs, and clinical outcomes. In fact, a good grading system is more important in its association with clinical symptoms than its high degree of inter-observer agreement or test-retest reliability. A confounding variable in this study is the non-recognition of lateral recess stenosis in the Schizas system 7-grade classification system particularly in grade A1,2,3,4, and B and is not included with this classification system. In the Lee classification system, the concept of lateral recess stenosis is omitted because the grading is based only on the effacement of the CSF space. Most studies about lumbar spinal stenosis focus on the LCCS. Failure to recognize presence of lateral recess stenosis is considered to be the main cause for failed back surgery on the lumbar spine [22]. In particular, in the case of grade A3 and A4 in the Schizas grading system are evaluated from Lee classification system to grade 0 stenosis, but in this case, the lateral recess stenosis might have occurred. Many studies have shown that radiological measurements or grading show poor clinical correlation [6–7, 23–24]. However, Mannion et al. reported that postoperative outcome was clearly related to the degree of preoperative radiological lumbar spinal stenosis [25]. So, the development of a reliable grading system that can reflect not only central canal stenosis but also lateral recess stenosis may increase the correlation between these grading systems and clinical symptom and treatment outcome. Finally, this is a single center study with a relatively small number of patients and validation using multi-center study with larger sample size is required to evaluate the reliability of these popular diseases.

## Conclusion

In conclusion, both Schizas and Lee MRI grading systems for LCCS are reliable grading systems, and can be used as a learnable method for both clinicians and radiologists.

## Supporting information

S1 File A supporting file includes; patient characteristics (sheet 1), 2^nd^ phase reading data for Shizas system (sheet 2), 2^nd^ phase reading data for Lee system (sheet 3), 3^rd^ phase reading data for Shizas system (sheet 4), and 3^rd^ phase reading data for Lee system (sheet 5). (XLSX)
